# Comparison of intravenous lidocaine and dexmedetomidine infusion for prevention of postoperative catheter-related bladder discomfort: a randomized controlled trial

**DOI:** 10.1186/s12871-019-0708-8

**Published:** 2019-03-18

**Authors:** S. Y. Li, H. Li, J. Ni, Y. S. Ma

**Affiliations:** Department of Anesthesiology, West China Second Hospital of Sichuan University, Key Laboratory of Birth Defects and Related Diseases of Women and Children, No.20, Section 3, Renmin Nanlu, Chengdu, China

**Keywords:** Catheter-related bladder discomfort, Lidocaine, Dexmedetomidine

## Abstract

**Background:**

Catheter-related bladder discomfort (CRBD) frequently occurs during recovery in patients who undergo intra-operative urinary catheterization. We conducted this study to compare the effect of intravenous lidocaine and dexmedetomidine infusion for preventing CRBD.

**Methods:**

120 patients undergoing elective open abdominal hysterectomy or hysteromyomectomy requiring urinary bladder catheterization were randomly allocated into three groups of 40 each. Group L received a 2 mg/kg lidocaine bolus followed by infusion of 1.5 mg/kg/h; Group D received a 0.5 μg/kg dexmedetomidine bolus followed by infusion of 0.4 μg/kg/h; Group C received a bolus and infusion of normal saline of equivalent volume. The incidence and different severity (mild, moderate, and severe) of CRBD were assessed on arrival in the postanaesthesia care unit at 0, 1, 2, and 6 h postoperatively.

**Results:**

The incidence of CRBD was significantly lower in Group L and Group D compared with Group C at 0, 1, and 2 h. However, there was no significant difference among the three groups regarding the different severity of CRBD at all time points. The requirement of rescue tramadol for CRBD was lower in group L and group D than in group C. The incidence of sedation was significantly higher in Group D compared to Group L and Group C, though no difference in other adverse effects was observed.

**Conclusions:**

Intravenous lidocaine and dexmedetomidine infusion reduced the incidence of CRBD as well as the additional tramadol requirement for CRBD, but had no effect on the different severity of CRBD.

**Trial registration:**

ChiCTR-INR-16009162. Registered on 5 September 2016.

## Background

Urinary catheterization is widely used during surgery, and the incidence of postoperative catheter-related bladder discomfort (CRBD) during recovery is as high as 47–90% [1, 2]. CRBD is similar to an overactive bladder (OAB), including urinary urgency and urinary frequency with or without urge incontinence, in addition to suprapubic pain [3]. Because CRBD is extremely distressing, it can increase postoperative pain and agitation, and thus often requires urgent treatment. The mechanism of CRBD is caused by involuntary contraction of the bladder, as mediated by muscarinic receptors, especially the subtype M_3_ receptor [4]. Various agents have been employed to manage CRBD, with varying degrees of success, including tolterodine, oxybutynin, butylscopolamine, paracetamol, gabapentin, pregablin, ketamine, tramadol, dexmedetomidine, darifenacin and solifenacin [5–18]. Although most of these drugs are helpful for managing CRBD, they also have many side effects, such as dry mouth, sedation, nausea, and vomiting. In addition, oxybutynin, tolterodine, gabapentin and pregablin are only preoperatively administered orally.

Dexmedetomidine is a selective a_2_-adrenoceptor agonist with analgesic, sympatholytic and sedative properties; it has been reported to have a beneficial effect in preventing CRBD by inhibiting the M_3_ receptor [16, 17]. Intravenous lidocaine infusion has analgesic and anti-inflammatory effects, reducing postoperative pain, nausea and vomiting, shortening hospital stay length, and promoting gastrointestinal function recovery [19, 20]. It is assumed that intravenous lidocaine has antimuscarinic properties [21]. We supposed that intravenous lidocaine infusion might be as useful as intravenous dexmedetomidine infusion for the prevention of CRBD. This study was designed to compare the effect of intravenous dexmedetomidine and lidocaine infusion for the prevention of CRBD and other adverse effects.

## Methods

This clinical trial was approved by the China Ethics Committee of Registering Clinical Trials, registered in the Chinese Clinical Trial Registry prior to patient enrollment. After obtaining written informed consent from all patients, we started this prospective, randomized, controlled, double-blind investigation. A total of 120 ASA class I–II patients, aged 18–60 years, who were scheduled for underwent elective open abdominal hysterectomy or hysteromyomectomy requiring a urinary bladder catheter were recruited. Patients with a history of bladder outflow obstruction, urinary tract infection, OAB, neurogenic bladder, chronic analgesic abuse, severe hepatic or renal disease, arrhythmia, allergy to lidocaine, and morbid obesity were excluded from this trial. Patients with intra-operative damage to the urinary tract or intestinal tract, massive haemorrhage, or operative time > 6 h were removed from this study.

Patients were randomly using computer-generated random numbers into three equal groups. Group L (lidocaine group) received a bolus of 2 mg/kg lidocaine infusion (10 min) before anesthesia induction, followed by an infusion of 1.5 mg/kg/h lidocaine. Group D (dexmedetomidine group) received a bolus of 0.5 μg/kg dexmedetomidine infusion (10 min) before anesthesia induction, followed by an infusion of 0.4 μg/kg/h dexmedetomidine. Group C (placebo control group) received a bolus and infusion of normal saline of equivalent volume. The study drugs were stopped at the end of the surgery. Monitoring of ECG, non-invasive blood pressure (BP), pulse oximetry (SPO_2_), end-tidal carbon dioxide (P_ET_CO_2_) and bispectral index (BIS) was performed for all patients. All patients received similar general anesthesia. Anesthesia was induced with midazolam 2 mg, sufentanil 3 μg/kg, and propofol 2 mg/kg, and intubation was facilitated with rocuronium 0.6 mg/kg. After induction of anesthesia, a 16F Foley urinary catheter (lubricated with lube) was insertered with 10 mL normal saline inflating the balloon to hold the catheter in bladder. Anesthesia was maintained with sevoflurane and additional sufentanil and rocuronium, while BIS was maintained at 40–60. At the end of the surgery, all patients received granisetron 3 mg and patient-controlled intravenous analgesia (PCIA) with sufentanil (0.5 μg/ml) and tramadol (4 mg/ml). Rocuronium was reversed with neostigmine when needed, but not for every patient. The extubation time was from drug withdrawal to full respiratory recovery time. Patients were transferred to the postanaesthesia care unit (PACU) after tracheal extubation for further recovery.

Bladder discomfort and pain were assessed on arrival at the PACU (0 h) and again at 1, 2, and 6 h by an anesthesiologist who was unaware of the patient grouping. Before surgery, patients were educated to differentiate CRBD from incisional or surgical pain. The severity of bladder discomfort was recorded as three grades: mild, revealed only on questioning; moderate, reported without questioning but not accompanied by any behavioral response; severe, stated on their own and followed by behavioral responses such as a strong verbal response, flailing limbs, and even attempting to pull out the urinary catheter [5, 6]. Rescue tramadol (50–100 mg) was administered when patients complained of moderate or severe CRBD, because tramadol has been shown to have an antimuscarinic effect [22]. We used the visual analog scale (VAS) score to evaluate postoperative pain which varied from 0 (no pain) to 10 (the worst imaginable pain). Rescue sufentanil 5 μg was administered when VAS > 4. Major adverse effects, such as nausea and vomiting, sedation, drowsiness, and dizziness, were recorded.

The sample size was estimated based on a preliminary experiment according to the incidence of CRBD in a range of 0.2 to 0.6 between three groups, with a = 0.05 and b = 0.10; 34 patients were needed in each group. Considering a dropout rate of 20%, 40 patients were included per group. The incidence and severity of CRBD, adverse effects, and additional tramadol requirements among the groups were assessed as percentage frequencies and tested by the chi-square test. The patients’ demographics and the time to extubation, duration of surgery, and intra-operative sufentanil requirement were evaluated by ANOVA. SPSS 17.0 was used for the statistical analysis, *P* < 0.017 was considered significant among the three groups and *P* < 0.05 between the two groups.

## Results

**Fig. 1:**
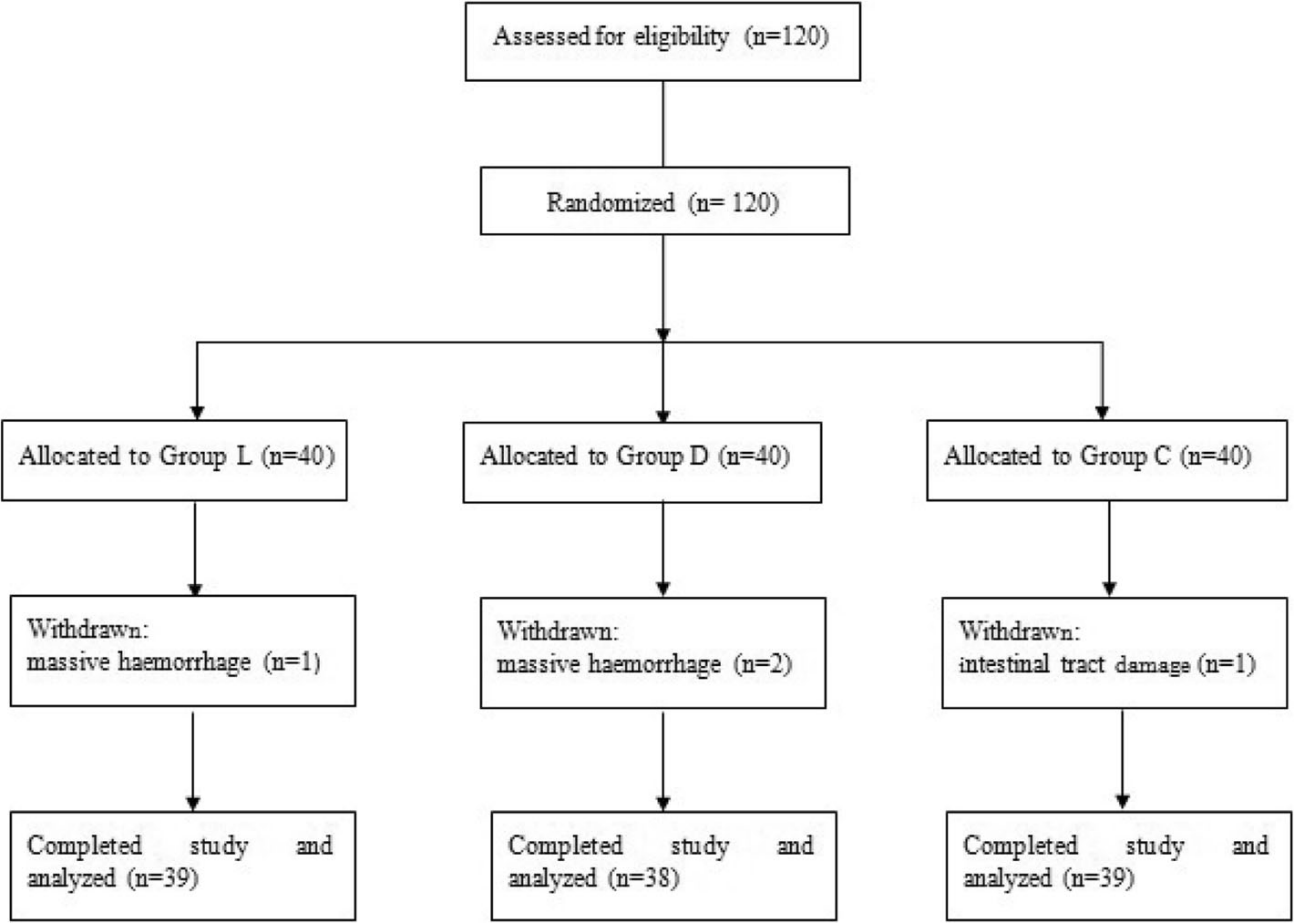
CONSORT flow diagram

A total of 120 patients were recruited in this study. Four patients were withdrawn for massive haemorrhage (3 patients) and intra-operative damage to the intestinal tract (1 patient). Ultimately, 116 patients (Group L = 39, Group D = 38, Group C = 39) were analyzed in this trial (Fig. 1). There were no significant differences in demographic data, duration of surgery, recovery time, extubation time or intra-operative sufentanil use among the three groups (Table 1).

**Table 1:** Patients characteristics and clinical data among the three groups

	Group L	Group D	Group C
Number of patients (*n*)	39	38	39
Age	45.0 ± 6.4	43.1 ± 5.8	44.4 ± 6.1
Weight (kg)	57.1 ± 7.9	56.8 ± 7.8	57.7 ± 7.1
Duration of surgery (Min)	124.1 ± 31.5	128.8 ± 34.7	119.5 ± 28.6
Time to extubation (Min)	12.6 ± 5.2	13.3 ± 4.3	13.3 ± 5.9
Intra-operative sufentanil requirement (ug)	23.8 ± 4.9	24.5 ± 7.0	25.6 ± 4.8

**Fig. 2:**
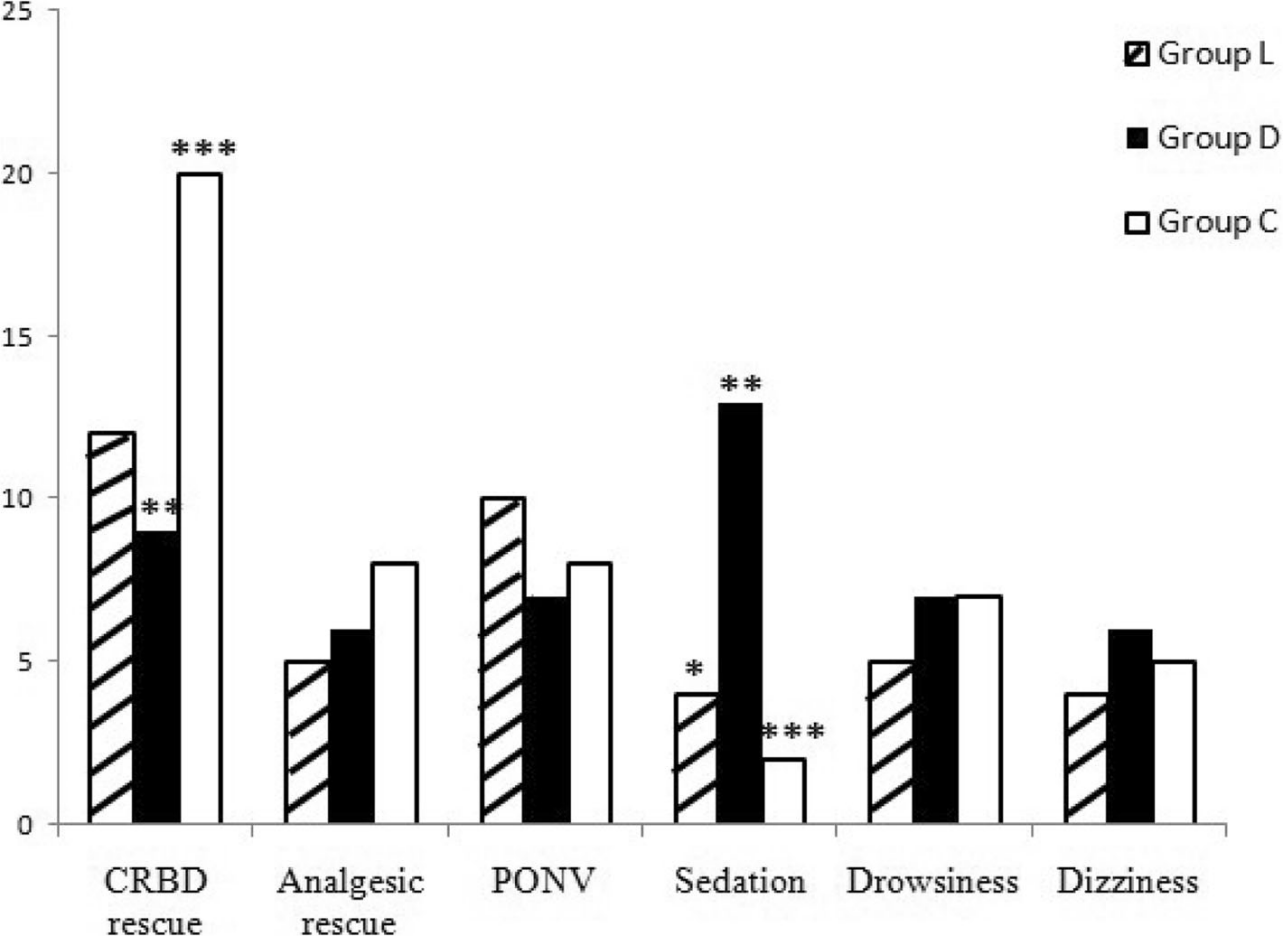
Adverse effects among the three groups (presented as numbers). ^&^*P* < 0.05, There is significant difference between group L and group D.*P < 0.05, There is significant difference between group D and group C. ^#^*P* < 0.05, There is significant difference between group L and group C

The incidence of CRBD was significantly lower in Group L compared to Group C at 0 h (*P* = 0.04), 1 h (*P* = 0.021) and 2 h (*P* = 0.023); and in Group D compared to Group C at 0 h (*P* = 0.004), 1 h (*P* = 0.004) and 2 h (*P* = 0.008); however, no difference between Group L and Group D was found. There was no significant difference among the three groups with respect to the different severity (mild, moderate, and severe) of CRBD at all time points (Table 2). The rescue tramadol requirement for CRBD was reduced in Group L (*P* = 0.02) and Group D (*P* = 0.012) compared to Group C, but there was no difference between Group L and Group D. Group D had a higher incidence of sedation than did Group L (*P* = 0.011) and Group C (*P* = 0.001). There were no differences in rescue analgesic requirement and other adverse effects (Fig. 2).

**Table 2:** Incidence and severity of CRBD presented as numbers (*n*)

Time	T0			T1			T2			T6		
Group	L	D	C	L	D	C	L	D	C	L	D	C
Incidence	17	13^*^	26^#^	18	15^*^	28^#^	13	11^*^	23^#^	7	5	9
Severity												
No	22	25^*^	13^#^	21	23*	11^#^	26	27^*^	16^#^	32	33	30
Mild	9	7	9	8	6	9	6	6	13	7	5	9
Moderate	8	6	13	10	9	17	7	5	10	0	0	0
Severe	0	0	4	0	0	2	0	0	0	0	0	0

*P < 0.05, There is significant difference between group D and group C. ^#^*P* < 0.05, There is significant difference between group L and group C

## Discussion

In this study, we found that intravenous lidocaine infusion was as effective as dexmedetomidine in reducing the incidence of CRBD and lessening the requirement of rescue tramadol.

CRBD caused by intra-operative urinary catheterization is a common and distressing complication, and it is a recognized risk factor for postoperative emergence agitation [23]. However, CRBD is frequently neglected in clinical practice. Severe CRBD is usually accompanied by behavioral responses, such as a strong vocal response, shaking of arms and legs, and pulling out the urinary catheter. Thus, management of CRBD might be helpful in reducing postoperative emergence agitation, decreasing the workload of medical staff and improving patient comfort.

It has been reported that certain surgeries, such as bladder surgery, prostate surgery, urinary tract surgery, and lower abdominal surgeries (such as obstetric and gynecological surgery), which can cause bladder spasms, are more likely to be associated with CRBD [1, 24, 25]. Consideration that the surgeries respected to the bladder or urethra can cause CRBD, in addition to the postoperative discomfort or pain originating from the surgical site, also seem to aggravate CRBD. In this investigation, patients undergoing open abdominal hysterectomy or hysteromyomectomy with the same-size catheter, we found a high incidence of CRBD up to 71.8%.

Dexmedetomidine is a selective a2-adrenoceptor agonist with analgesic, sympatholytic and sedative properties, without respiratory depression. Some studies have shown that dexmedetomidine decreases the incidence and severity of CRBD. The drug acts by M_3_ receptors [16, 17, 26]. In our study, we found that dexmedetomidine reduced the incidence of CRBD as well as the additional tramadol requirement for CRBD but not the different severity (mild, moderate, and severe) of CRBD. This difference may be explained by female gender and surgery properties in our study. Considering that dexmedetomidine was stopped at the end of the surgery, the postoperative sedative was higher in the dexmedetomidine group than in the other groups. The higher incidence of sedation might be also the reason for the low incidence of CRBD.

Intravenous lidocaine infusion has been demonstrated to decrease intra-operative opioid requirements, reduce postoperative pain, nausea and vomiting, ameliorate postoperative ileus, shorten the length of hospital stay [19, 20]. The mechanisms of systemically managing lidocaine include antagonism of NMDA receptors, anti-inflammatory effects on histamine, prostaglandins and kinins, suppressing C-afferent neuronal activity and neural excitability in dorsal horn neurons, and inhibiting spinal visceromotor neurons [19, 20, 27]. In this study, we found that intravenous lidocaine infusion had the similar effect of intravenous dexmedetomidine infusion on preventing of CRBD. We suggest that it may act though an anti-inflammatory effect and blockade of C-afferent neuronal activity.

There were some limitations in this study. First, since it was well known that lidocaine infusions and dexmedetomidine infusions could modulate all sorts of postoperative discomfort, but we did not evaluate the level of discomfort related not only to bladder discomfort, but also surgical pain or other discomfort. Second, this study was limited to the period of the drug metabolism in the immediate postoperative period, and failed to address patient bladder discomfort beyond 6 h. Moreover, the severity of CRBD was recorded as three grades as mild, moderate, and severe which various descriptors were applied to these levels. But, if we used visual analog scale (VAS) or verbal rating scale (VRS) to evaluate CRBD, the severity of CRBD might had significant difference among the three groups in this study. Furthermore, we did not evaluate the efficacy of all types of surgery, different surgeries have different degrees of interference. Therefore, further studies are warranted.

## Conclusion

In conclusion, intravenous lidocaine and dexmedetomidine infusion reduced the incidence of CRBD as well as the additional rescue requirement for CRBD but not its different severity (mild, moderate, and severe) of CRBD in patients undergoing open abdominal hysterectomy or hysteromyomectomy.

## Abbreviations

ANOVA: One-way analysis of variance
BIS: Bispectral index
CRBD: Catheter related bladder discomfort
OAB: Overactive bladder
PACU: Post-anaesthesia care unit
PCIA: Patient-controlled intravenous analgesia
P_ET_CO_2_: End-tidal carbon dioxide
SPO_2_: Pulse oximetry
